# SARS-CoV-2 molecular diagnostic point-of-care testing based on loop-mediated isothermal amplification: A prospective, single-center validation study

**DOI:** 10.1016/j.heliyon.2023.e14564

**Published:** 2023-03-15

**Authors:** Sung Hun Moon, Sang-Chul Kim, Byung Woo Kim, Gwan-Jin Park, Hyun-Seok Chai, Young Min Kim, Hee Sung Kim, Hee Sue Park

**Affiliations:** a Department of Emergency Medicine, Chungbuk National University Hospital, 776, 1st Sunhwan-ro, Seowon-gu, Cheongju-si, Chungcheongbuk-do 28646, Republic of Korea; b Department of Emergency Medicine, College of Medicine, Chungbuk National University, 1 Chungdae-ro, Seowongu, Cheongju-si, Chungcheongbuk-do 28646, Republic of Korea; c Department of Paramedic Science, Korea National University of Transportation, 61, Daehak-ro, Jeungpyeong-gun, Chungcheongbuk-do, 27909, Republic of Korea; d Department of Internal Medicine, Chungbuk National University Hospital, 776, 1st Sunhwan-ro, Seowon-gu, Cheongju-si, Chungcheongbuk-do 28646, Republic of Korea; e College of Medicine and Medical Research Institute, Chungbuk National University, 1 Chungdae-ro, Seowongu, Cheongju-si, Chungcheongbuk-do 28646, Republic of Korea; f Department of Laboratory Medicine, Chungbuk National University Hospital, 776, 1st Sunhwan-ro, Seowon-gu, Cheongju-si, Chungcheongbuk-do 28646, Republic of Korea; g Department of Laboratory Medicine, College of Medicine, Chungbuk National University, 1 Chungdae-ro, Seowongu, Cheongju-si, Chungcheongbuk-do 28646, Republic of Korea

**Keywords:** COVID-19, Point-of-care testing, LAMP Assay, Viral load

## Abstract

**Objectives:**

Rapid and accurate severe acute respiratory syndrome coronavirus 2 (SARS-CoV-2) diagnostic tests are crucial for controlling the spread of infections in emergency settings. This study evaluated the diagnostic accuracy of a point-of-care (POC) test based on loop-mediated isothermal amplification (LAMP) that produces rapid results within 30 min.

**Methods:**

We prospectively included adult patients (age >19 years) who were diagnosed with SARS-CoV-2 infection within the last 3 days and symptomatic patients who had visited the emergency room. Posterior nasopharyngeal (PNP) swabs and throat swabs collected by physicians were used to test the sensitivity, specificity, positive predictive value (PPV), negative predictive value (NPV), accuracy, and Cohen's Kappa coefficient (k) of the POC index and reference reverse transcription quantitative polymerase chain reaction (RT-qPCR) test devices.

**Results:**

Of the 352 participants, 102 (29.0%) tested positive via the RT-PCR-based reference test device; the RT-LAMP-based POC test had a sensitivity of 70.6% and specificity of 98.0%, with 93.5% PPV, 89.1% NPV, 35.5% PLR, and 3.4% NLR. Cohen's k correlation of results from the two devices was 0.74. The cycle threshold value between the positive and negative POC test results differed (17.6 vs. 24.6, *p* < 0.001).

**Conclusions:**

The RT-LAMP POC test in the emergency medical setting has a fair predictive value in high viral load cases in terms of infectivity.

## 1 Introduction

The severe acute respiratory syndrome coronavirus 2 (SARS-CoV-2) infection outbreak was first reported from Wuhan, People's Republic of China, on December 12, 2019. Following the World Health Organization's (WHO) declaration on March 11, 2020, that the coronavirus disease (COVID-19) was a pandemic caused by SARS-CoV-2, approximately 0.6 billion confirmed cases and more than 6 million SARS-CoV-2-related deaths had been recorded worldwide by August 29, 2022 [1]. In addition to identification of patients, early contact tracing, and critical decision-making on public health policies, rapid and accurate diagnostic tests for SARS-CoV-2 are essential in the field of emergency medicine to minimize the spread of SARS-CoV-2 [2,3].

Reverse transcription quantitative polymerase chain reaction (RT-qPCR)-based nucleic acid amplification test (NAAT) is the most sensitive method to date and is the gold standard method for the diagnosis of SAR-CoV-2 infection. Samples for SARS-CoV NAAT are obtained from the nasopharynx, oropharynx, nasal mid-turbinate, or anterior nares by swabbing, and NAAT requires 3.5–4.0 h for RNA extraction, complementary DNA synthesis, and amplification of the target nucleic acid [4]. Therefore, NAAT commonly requires laboratory-based centralized testing, which is time consuming and needs specialized equipment and trained personnel [5]. Owing to the insufficiency of isolation rooms, the length of emergency room stay is prolonged—this further extends the prehospital duration for patients with suspected SARS-CoV-2 infection who have to be transported to appropriate hospitals [6].

Point-of-care (POC) testing for the diagnosis of respiratory virus infections is cost-effective, and thus the faster, inexpensive, equally sensitive POC testing has attracted the spotlight for SARS-CoV-2 diagnosis. Many POC-based rapid antigen tests are being used for SARS-CoV-2 detection [7]. Although specificity is high, sensitivity of POC testing varies from 15% to 95%, depending on symptom status, test timing, and test brands [8]. Therefore, POC testing can be used for screening of patients for SARS-CoV-2 infection. The isothermal nucleic acid amplification method has similar specificity as that provided by RT-PCR, and owing to high sensitivity, flexibility, and short swab-to-result time with the omission of the RNA extraction process, this test has a similar widespread application as that of a novel POC test [9].

The loop-mediated isothermal amplification (LAMP) test is designed to be capable of detecting even a few copies of target nucleic acid sequences under isothermal conditions at temperatures between 60 and 65 °C; thus, SARS-CoV can be detected using the LAMP test with the help of specifically designed primer sets and reverse transcriptases [10]. The nucleic acid amplification products can be measured real-time based on colorimetric, fluorimetric, and turbidity changes resulting from to pH changes and/or the byproducts formed [[11], [12], [13]] Skipping the thermocycling step and the real-time measurement of the levels of amplification products contribute to the short testing time from sampling to result readout, enabling the RT-LAMP test to serve as a personal POC diagnostic because the results can also be shared via smartphone applications [14]. Thus, RT-LAMP-based POC testing can be used in offices, airports, schools, ambulances, and emergency rooms, if the accuracy is guaranteed. The diagnostic accuracy needs to be tested with an effectiveness study for a RT-LAMP based POC testing with a short result time of less than 30 min for screening suspected COVID-19 patients who visit the emergency room. This study aimed to compare the diagnostic accuracy of an index test device, a RT-LAMP based POC testing device with a reference test device (RT-PCR) for SARS-CoV-2 detection.

## 2 Materials and methods

### 2.1 Study design and setting

This prospective, single-center, point-of-care validation study was performed between October 2021 and March 2022 at the emergency medical center of XXX Hospital (XXXX), XXX, XXXX. The XXX is a 711-bed tertiary-care University Hospital with approximately 40,000 emergency room visits each year. In response to the COVID-19 outbreak, the XXXX remodeled the emergency center to create a designated negative-pressure isolation area (8 beds) to spatially separate patients presenting with fever or respiratory symptoms. At the entrance of the hospital, patients who had had close contact with a confirmed COVID-19 case within the past 14 days or showed symptoms of SARS-CoV-2 infection were directed to the designated isolation area for diagnostic tests. Next, patients who tested negative via a RT-qPCR-based reference test device (AccuPower® RV1 Multiplex Kit; Bioneer; South Korea) were allowed into the normal emergency department (ED) area.

### 2.2 Participants

We included adult patients (age ≥19 years) who were diagnosed with SARS-CoV-2 in the normal ED area or in other hospitals within the last 3 days and patients who visited the ED with any of the 11 common signs and symptoms noted by the U.S. Centers for Disease Control and Prevention as follows: fever or chills, cough, dyspnea, fatigue, muscle pain, headache, new loss of taste or smell, sore throat, congestion or runny nose, nausea or vomiting, and diarrhea. We excluded patients whose results via the index test device were invalid and those who were diagnosed with and treated for SARS-CoV-2 within the preceding 7 days.

### 2.3 Specimen collection and laboratory testing

A COVID-19 test with an index test device was performed by physicians. The index test device (1 copy™ COVID-19 MDx Kit Professional, 1 drop, South Korea) used in our analysis is a POC molecular diagnostic test device that generates results within 30 min from sampling to result readout. Based on RT-LAMP, an isothermal nucleic acid amplification method, the device requires neither RNA extraction nor thermocycling. For the reference test device, nucleic acid extraction was performed automated Exiprep TM48DX equipment (Bioneer, Korea). And RT-PCR SARS-CoV-2 test were performed using AccuPower SARS-CoV-2 assay (Bioneer, Korea) according to the manufacturer's protocol in CFX96TM Dx System (Bio-Rad, USA).

Test samples were obtained in a sample collection room or at the bedside in the form of posterior nasopharyngeal (PNP) swabs and/or throat swabs for the index and reference test device. The samplings for each participant were performed by the same medical staff in order to minimize the irregularity in sampling. Training on sampling and handling the POC molecular diagnostic test devices was conducted before the study was initiated. PNP samples were tested immediately after collection by trained medical staff. After running the application on a smartphone and warming the index device, the tubes were inserted into the index device. After reaction and amplification, the results were interpreted according to the manufacturer's instructions as positive, negative, or invalid.

Combined PNP and/or throat swabs were diluted in a sample tube with standard genetic material. For RT-PCR laboratory confirmation, the sample tube was transported to the certified laboratory of the study-center hospital in a sterile box that was double-bagged with a commercially manufactured vinyl bag. Automated RNA extraction with the reference device was performed after dividing the sample into cartridges of up to 48 samples. Samples were screened for SARS-CoV-2 RNA through qualitative RT-PCR, which targeted three regions that had conserved sequences: E gene (envelope protein gene), RdRp gene (RNA-dependent RNA polymerase gene) in the open reading frame ORF1ab region, and N gene (nucleocapsid protein gene). The primer set of the index test device was designed to detect N-gene-specific sequences of SARS-CoV-2. Samples with a SARS-CoV-2 RT-qPCR cycle threshold (Ct) value less than 41 for all three regions (E gene, RdRp gene, or N gene) were considered to have a positive result.

### 2.4 Sample size and statistical analysis

When the prevalence of SARS-CoV-2 infection was estimated at 30% during the COVID-19 pandemic (Omicron), a minimum sample size of 357 patients, including 107 patients with the disease, would be required to achieve the minimum power of 80% for detecting a change in the percentage value of sensitivity of a diagnostic test from 0.70 to 0.90, based on a target significance level of 0.05.

We used Excel (ver. 2013, Microsoft®, Santa Rosa, CA, USA) to complete all data analysis. Continuous variables are presented as mean ± standard deviation and median (interquartile range, IQR), whereas categorical variables are presented as frequencies and relative proportions. For comparisons of continuous variables, we used the nonparametric Mann–Whitney *U* test owing to the small sample size; for comparisons of categorical variables, we performed either the chi-square or Fischer's exact test, depending on the applicability.

The sensitivity, specificity, positive predictive value (PPV), negative predictive value (NPV), accuracy, Kappa coefficient (Kappa), and 95% CI were calculated to determine the predictive validity of the index test device and the level of agreement with the reference test device, which is considered the gold standard method for this validation study.

## 3 Results

### 3.1 Participant demographic

**Fig. 1:**
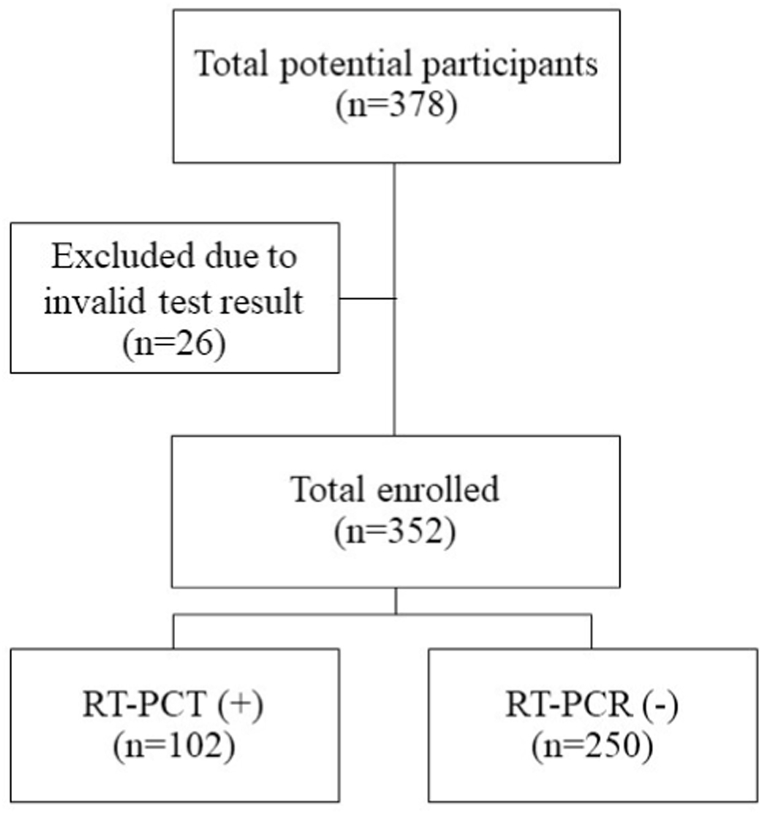
Flow diagram. RT-PCR: Reverse Transcription Polymerase Chain Reaction.

Of the 378 potential participants who were screened, 352 met the eligibility criteria for inclusion in the clinical trial and 26 were excluded whose results via the index test device were invalid (Fig. 1). Table 1 shows the baseline characteristics of the participants. The median age was 57.0 (IQR 33.0–74.8) years, and 48.3% were men. A total of 102/352 (29.0%) tested positive via the standard RT-qPCR test. Among the reasons for POC testing, the most common was fever symptom (45.2%). The proportions of cases with epidemiologic relation, sore throat, and gastrointestinal symptoms were higher in the positive RT-qPCR group. The median duration of symptoms before visiting the study hospital was 11 (IQR 2–48) hours in this cohort.

**Table 1:** Baseline characteristics of the participants.

Characteristics	Total N = 352	RT-PCR (+) n = 102	RT-PCR (−) n = 250	*p*-value
Age, years				
Median (IQR)	57.0 (33.0–74.8)	58.0 (33.8–72.3)	56.0 (32–8)	0.648
**Sex**, n (%)				
Male	170 (48.3)	46 (45.1)	124 (49.6)	0.443
Female	182 (51.7)	56 (54.9)	126 (50.4)	
**Cause of POC testing**, n (%)				
Fever	159 (45.2)	19 (18.6)	140 (56.0)	**<0.001**
Respiratory symptom	89 (25.3)	24 (23.5)	65 (26.0)	
Epidemiologic relation	50 (14.2)	24 (23.5)	26 (10.4)	
Sore throat	20 (5.7)	15 (14.7)	5 (2.0)	
Gastrointestinal symptom	7 (2.0)	5 (4.9)	2 (0.8)	
Others	27 (7.7)	15 (14.7)	12 (4.8)	
Body temperature (°C)				
Median (IQR)	37.0 (36.5–37.8)	37.0 (36.6–37.6)	37.2 (36.5–37.8)	0.453
**Duration of symptoms** (hours)				
Median (IQR)				
11 (2–48)	10.5 (2–48)	11 (2–48)	0.935	
**Result of POC testing**, n (%)				
Positive	77 (21.9)	72 (70.6)	5 (2.0)	**<0.001**
Negative	275 (78.1)	30 (29.4)	245 (98.0)	
**CT value**				
Median (IQR)				
19.2 (15.4–24.9)				
**Clinical outcome**, n (%)				
Discharge	202 (57.4)	55 (53.9)	147 (58.8)	0.155
Admission	142 (40.3)	42 (41.2)	100 (40.0)	
Transfer	4 (1.1)	3 (2.9)	1 (0.4)	
Expire	4 (1.1)	2 (2.0)	2 (0.8)	

Statistically significant values in bold. N/n: Number; RT-PCR: Reverse Transcription Polymerase Chain reaction; IQR: Inter Quartile Range; POC: Point Of Care; CT: Cycle Threshold.

Table 2 shows the validation results of the POC test versus the laboratory-based RT-qPCR test. The sensitivity of the POC test was 70.6% (95% CI 61.7–79.4) and the specificity was 98.0% (95% CI 96.3–99.7) with 93.5% PPV, 89.1% NPV, 35.5% PLR, and 3.4% NLR. Accuracy was 90.1%, and the Cohen's k correlation between the two tests was 0.74 (95% CI 0.66–0.82).

**Table 2:** Validation results of the POC test.

	Formula	Value	95% CI
Sensitivity	72/102	70.6%	61.7–79.4
Specificity	245/250	98.0%	96.3–99.7
Positive predictive value	72/77	93.5%	88.0–99.0
Negative predictive value	245/275	89.1%	85.4–92.8
Positive likelihood ratio	0.71/(1 − 0.98)	35.5%	14.7–84.8
Negative likelihood ratio	0.98/(1 − 0.71)	3.4%	22.2–40.6
Accuracy	317/352	90.1%	86.9–93.2
Kappa coefficient		0.740	0.66–0.82

POC: Point Of Care; CI: Confidence Interval.

Table 3 shows the baseline characteristics of participants with positive RT-qPCR test results. The median Ct value between positive POC and negative POC test results differed (17.6 vs. 24.6, p < 0.001). The median age was 58.0 (IQR 33.0–74.8) years, and 45.1% of this sub cohort were men. As the Ct value of the positive reference test device increased, the sensitivity of the index test device decreased (Table 4, p < 0.001).

**Table 3:** Baseline characteristics of SARS-CoV-2 RT-PCR-positive participants.

Characteristics	Total N = 102	POC testing (+)	POC testing (−)	p-value
CT value				
Median (IQR)				
	19.2 (15.4, 24.9)	17.6 (14.9, 21.8)	24.6 (20.0, 31.6)	<0.001
Age, year				
Median (IQR)	58.0 (33.8–72.3)	51.0 (33.3–72.0)	61.5 (40.8–74.3)	0.256
Sex, n (%)				
Male	46 (45.1)	33 (45.8)	13 (43.3)	0.831
Female	56 (54.9)	39 (54.2)	17 (56.7)	
Indication for POC, n (%)				
Fever	19 (18.6)	12 (16.7)	7 (23.3)	0.266
Respiratory symptom	24 (23.5)	15 (20.8)	9 (30.0)	
Epidemiologic relationship	24 (23.5)	17 (23.6)	7 (23.3)	
Sore throat	15 (14.7)	13 (18.1)	2 (6.7)	
Gastrointestinal symptom	5 (4.9)	5 (6.9)	0 (0.0)	
Others	15 (14.7)	10 (13.9)	5 (16.7)	
Body temperature (°C)				
Median (IQR)	37.0 (36.6–37.6)	37.0 (36.7–37.5)	37.0 (36.4–37.7)	0.939
Duration of symptoms (hours)				
Median (IQR)	10.5 (2.48)	11 (3, 23)	10 (1, 72)	0.359
Clinical outcome, n (%)				
Discharge	55 (53.9)	40 (55.6)	15 (50.0)	0.770
Admission	42 (41.2)	27 (37.5)	15 (50.0)	
Transfer	3 (2.9)	3 (4.2)	0 (0.0)	
Expire	2 (2.0)	2 (2.8)	0 (0.0)	

Statistically significant values in bold. SARS-CoV-2: Severe Acute Respiratory Syndrome Coronavirus 2; RT-PCR: Reverse Transcription Polymerase Chain Reaction; N/n: Number; POC: Point Of Care; CT: Cycle Threshold; E: Envelope Protein; IQR: Inter Quartile Range; RdRp: Ribonucleic Acid-dependent Ribonucleic acid Polymerase.

**Table 4:** Comparison of sensitivity according to CT value among SARS-CoV-2 RT-PCR-positive participants.

CT value	Total N = 102, n (%)	POC testing (+) n = 72, n (%)	POC testing (−) n = 30, n (%)	Sensitivity
≤15	21 (20.6)	19 (26.4)	2 (6.7)	90.5
≤20	55 (53.9)	48 (66.7)	7 (23.3)	87.3
≤25	77 (75.5)	62 (86.1)/	15 (50.0)	80.5
≤30	90 (88.2)	70 (97.2)/	20 (66.7)	77.8
≤36	102 (100.0)	72 (100.0)	30 (100.0)	70.6

CT: Cycle Threshold, SARS-CoV-2: Severe Acute Respiratory Syndrome Coronavirus 2; RT-PCR: Reverse Transcription Polymerase Chain Reaction; N/n: Number, POC: point of care.

**Fig. 2:**
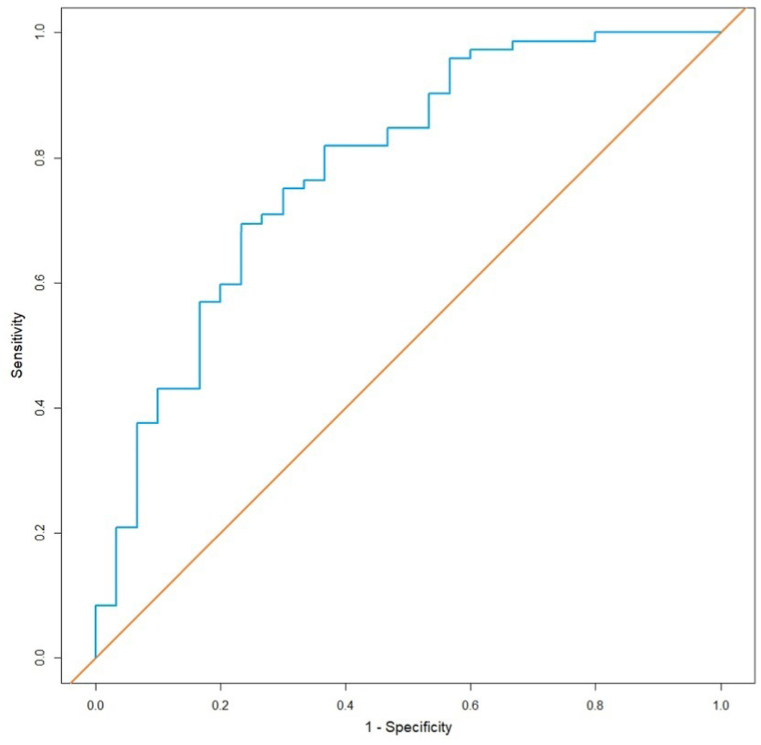
Receiver operating characteristic curve of cycle threshold (Ct) value to predict positive POC test result. Area under the curve determined using qualitative RT-PCR is 0.784 (95% confidence interval 0.682–0.885) with a cutoff Ct value of 20.6 (69.4% sensitivity, 76.7% specificity, 48.9% PPV, and 12.3 NPV).

Receiver operating characteristic (ROC) curves were generated for the positive results of the index test device and compared with the positive results of the reference test device (Fig. 2). The resulting area under the curves were 0.784 (95% CI 0.682–0.885, 69.4% sensitivity, 76.7% specificity, 48.9% PPV, and 12.3% NPV) for the reference test device, and we demonstrated a fair prediction value of a positive POC test result with 20.6 C t value.

## 4 Discussion

We performed a prospective clinical validation trial for 6 months to evaluate the detection of SARS-CoV-2 with a RT-LAMP POC based index test in comparison with the laboratory-based standard RT-qPCR test in suspected COVID-19 patients who presented to the ED. The sensitivity of the index test device was 70.6%, and the specificity was 98.0%. The PPV was high (93.5%) because the study period included the peak incident point of the Omicron variant-driven surge in the epidemic. The Ct value of 21 in the laboratory-based standard RT-qPCR was the cutoff value used to predict a positive result on the index test.

In terms of the sampling process of the index test device, the sensitivity results using crude samples such as in the present study could be lower than those with purified RNA samples owing to the additional process of sampling of specimens and preservation of unstable RNA virus sampling. According to the meta-analysis of RT-LAMP POC tests for human coronavirus, the average sensitivity in 15 studies that used crude samples was 0.78 (95% CI: 0.65–0.87) and specificity was 0.96 (CI: 0.95–0.99), which are similar to the results of this study. In contrast, the average sensitivity in the results of 26 studies using purified RNA samples was 0.94 (95% CI: 0.90–0.96) and specificity was 1.00 (95% CI: 0.99–1.00) [15].

Given the rapid spread of SARS-CoV-2 and its associated morbidity and mortality, effective POC diagnostic tools, the efficacy of which should be comparable to that of the gold standard RT-PCR test for detecting SARS-CoV-2, are required. Instead of RT-PCR, which is time-consuming, expensive, and complicated, and hence, scarcely meets the need for large-scale and POC screening, several alternatives, such as serology, rapid antigen, and RT-LAMP tests, have become available [16,17].

Since serology tests detect the presence of anti-SARS-CoV-2 antibodies in the plasma or serum of infected individuals, they provide only indirect evidence of infection 1–2 weeks after the onset of symptoms and are thus, sensitive for the later and recovery stages of the infection; accordingly, these tests are best used for surveillance, and not for screening [18,19]. Antibody tests can play a supplementary but indispensable role in the diagnosis of suspected cases with negative viral RNA test or past infection history of COVID-19 and for surveillance and epidemiological assessment at a population level [[20], [21], [22], [23]].

Rapid antigen tests detect the presence of specific SARS-CoV-2 antigens in respiratory samples; these tests show an easy accessibility and are associated with a short wait time (about 15 min) from the sampling step to the result collection step. Antigen tests have been proven to be more affordable than RT-PCR and more sensitive during the infectious period of the disease [24]. However, rapid antigen tests showed a clinical sensitivity of around 65% (58% in some cases); their accuracy is thus, lower than that of the RT-LAMP assay. For testing clinical samples, the LAMP assays showed a very high specificity (80%–100%) and sensitivity (73%–100%), using repeated RT‐PCR as a reference [10,25].

The RT-LAMP POC testing can be a decentralized method for decreasing the ED stay for suspected COVID-19 patients, which will be related to rapid turnover time, improvement of infection control, overcrowding, and patient flow in the ED [26,27]. Current RT-LAMP assays are one to two times less sensitive than RT‐qPCR, which result in substantial false-negative rates [28]. The limited sensitivity of the RT-LAMP POC based index test might constitute the main obstacle to the reliable usage of this device in the field as a POC.

The sensitivity of the RT-LAMP POC based index test device for COVID-19 depends on the viral load in specimens [15]. A Ct value of ≤25 was defined as a high viral load in previous studies and is higher than the Ct cutoff of 21 used in this study [8]. The average Ct value of the samples that tested negative on our index test device was 25. This means that crude samples with a Ct value above 25 might show a false-negative result in the POC test. However, Bullard et al. reported that patients with RT-PCR Ct values greater than 24 or a duration greater than 8 days between symptom onset and testing may have low infectivity. Therefore, our index test device has usefulness in the screening capacity of COVID 19 when considering infectivity and not merely sensitivity.

This study had a number of limitations. First, identical and accurate swabbing techniques could not be applied for all patients. We reasonably speculate that it might be related with invalid test results (Fig. 1, n = 26) or affect unsatisfied sensitivity of index test device. Nasopharyngeal swabs should be performed correctly to enhance absorption of secretions useful for diagnosis at reaching the nasopharynx. However, nasopharyngeal swabs may be impaired by reduced patient cooperation at the bedside of ED environment. The negative results of the index test for symptomatic patients with a high viral load might be caused by improper sampling of specimens, anatomic conditions, sampling technique, and inadequate preservation of samples [4,29]. Therefore, more correct training and education for medical staffs is needed to make diagnostic procedure more accurate and, especially in the case of PNP swab. Second, we could not evaluate the diagnostic accuracy of the test device according to symptom status and duration from symptom onset because most of our participants were symptomatic, with a median duration of symptoms being present for 11 h. The average sensitivity of the POC test for symptomatic participants was higher than that for asymptomatic participants (72.0%, vs. 58.1%).

## 5 Conclusion

The RT-LAMP POC based index test, which provides rapid results within 30 min, did not meet WHO's criteria of sensitivity of ≥80% for a rapid diagnostic test. However, this test showed a fair predictive value for SARS-CoV-2-positive patients with high viral load in terms of infectivity. Therefore, if the accurate diagnostic procedure is assured, the RT-LAMP POC based index test device will be useful for screening suspected COVID-19 patients to quickly facilitate patient isolation in the COVID-19 pandemic situation.

## Author contribution statement

Sang-Chul Kim: Conceived and designed the experiments; Performed the experiments; Wrote the paper.

Sung Hun Moon: Performed the experiments; Wrote the paper.

Byung Woo Kim: Analyzed and interpreted the data; Wrote the paper.

Gwan-Jin Park: Analyzed and interpreted the data.

Hyun-Seok Chai; Young Min Kim; Hee Sue Park: Contributed reagents, materials, analysis tools or data.

Hee Sung Kim: Performed the experiments.

## Funding statement

This work was supported by 10.13039/501100002461Chungbuk National University BK21 program (2021).

## Data availability statement

Data will be made available on request.

## Declaration of interest's statement

The authors declare no competing interests.
